# Effect of oocyte activation with calcium ionophore on ICSI outcomes in teratospermia: A randomized clinical trial 

**Published:** 2013-11

**Authors:** Maryam Eftekhar, Sima Janati, Mozhgan Rahsepar, Abbas Aflatoonian

**Affiliations:** 1 *Research and Clinical Center for Infertility, Shahid Sadoughi University of Medical Sciences, Yazd, Iran.*; 2 *Department of Obstetrics and Gynecology, Dezful University of Medical Sciences, Dezful, Iran.*

**Keywords:** *Intracytoplasmic sperm injection*, *Calcium ionophore*, *Oocyte activation*, *Fertilization*

## Abstract

**Background:** Chemical activation is the most frequently used method for artificial oocyte activation (AOA), results in high fertilization rate.

**Objective: **This prospective, randomized, unblinded, clinical study aimed to evaluate the efficiency of oocyte activation with calcium ionophore on fertilization and pregnancy rate after intracytoplasmic sperm injection (ICSI) in infertile men suffer from teratoospermia.

**Materials and Methods:** Thirty eight women with teratoospermic partner underwent ICSI with antagonist protocol. A total of 313 metaphase II (MII) oocytes were randomly divided into two groups: In the oocytes of the control group (n=145), routine ICSI was applied. Oocytes in the AOA group (n=168) immediately after ICSI, were entered in culture medium supplemented with 5 µΜ calcium ionophore (A23187) for 5 minutes and then washed at least five times with MOPS solution. In both groups, the fertilization was evaluated 16-18 hours after ICSI.

**Results:** The number of fertilized oocytes and embryos obtained were significantly different between two groups (p=0.04). There was no significant difference between the two studied groups regarding the fertilization and cleavage rate (95.33% vs. 84.4%, p=0.11; and 89.56% vs. 87.74%, p=0.76, respectively). Implantation rate was higher in AOA group than in control group, but the difference was not significant (17.64% vs. 7.4%, p=0.14). No significant differences were observed in chemical and clinical pregnancy rate between groups (47.1% vs. 16.7%, p=0.07; and 41.2% vs. 16.7%; p=0.14, respectively).

**Conclusion:** We didn’t find significant difference in the implantation, fertilization, cleavage and pregnancy rates between the two groups but could significantly increase the number of fertilized oocytes and embryos obtained. Finally oocyte activation with calcium ionophore may improve ICSI outcomes in infertile men suffer from teratoospermia. Further study with more cases can provide greater value.

## Introduction

Infertility influences one in seven couples generally and is a growing problem worldwide (1). Assisted reproductive techniques are currently accountable for up to 7% of childbirths in developed countries    (2). Intracytoplasmic sperm injection (ICSI) has been developed as one of the best efficient therapeutic approach for male factor infertility (3). Whilst some crucial stages in normal fertilization are bypassed in ICSI, the average fertilization rate remains at 60-70% (4, 5). Fertilization failure after ICSI can be defined by imperfections in oocyte, sperm, as well as, ICSI technique (5, 6). After ICSI, while more than 80% of oocytes have spermatozoa fertilization failure is probably due to lack of activation of the oocyte, or an incapability of the oocyte to decondense the sperm (7-9). 

Oocyte activation is one of the first events that occurs at fertilization and characterized by two basic molecular events (5, 10): an elevation of intracellular calcium concentration, due to the initial release from the endoplasmic reticulum which occurs 1-3 minutes after binding of the sperm to the oolemma, and a transient calcium elevation in oocyte as a main trigger of meiotic resumption during fertilization (6). Many investigators have tried different protocols for artificially activating oocytes to overcome fertilization failure after ICSI, including chemical, electrical and mechanical methods (4, 6). Among them, chemical activation is the most commonly used method for artificial oocyte activation (4). 

Many chemical substances are known to induce an intracellular calcium surge and subsequently activate the oocytes, such as calcium ionophore (A23187), ethanol, ionomycin, puromycin, strontium chloride, probol ester and thimerosal (5, 6). Several experiments have revealed that the artificial oocyte activation with calcium ionophore led to an increase in intracellular free calcium, mimicking physiologic mechanisms that cause oocyte activation (11, 12). Therefore, this study was designed to investigate the effect of artificial oocyte activation with calcium ionophore (A23187) on fertilization, cleavage and pregnancy rate after ICSI in infertile men suffering from teratoospermia. 

## Materials and methods

This study was approved by Ethics Committee of Yazd Research and Clinical Center for Infertility, Shahid Sadoughi University of Medical Sciences, Yazd, Iran.

**Patient Selection**

This prospective, randomized, unblinded, clinical trial was performed on 38 women with teratoospermic partner (normal morphology <14%) undergoing to ICSI cycles in Yazd Research and Clinical Center for Infertility, during the April to December 2012. Teratoospermia in men confirmed based on their semen analysis. The patients were randomly allocated into two groups using a computer based randomization list. Written informed consent was obtained from all of the couples prior to the study. The women with aged more than 40, FSH ≥12 IU/L, immature oocyte, oocyte deformity were eliminated from this study.

**Ovarian stimulation and ovum pickup**

Controlled ovarian stimulation was performed by using the antagonist protocol. All patients received low-dose oral contraceptive pills (Ocp LD) (30 μg Ethinyl Estradiol and 0.3 mg Norgestrel, Aburaihan Pharmaceutical Co., Tehran, Iran) was began on 2^nd^ day of the cycle then discontinued till menstruation occurred. Once menses began, gonadotropin stimulation by Gonal-F (Gonal-F, Serono, Italy) was started from the 2nd day of the menstrual cycle .The starting dose of gonadotropin was 150-300 mIU/d, according to the patient’s age and body weight. 

Monitoring was started on day 7 of stimulation and dose of gonadotropin was adjusted based on the serum E_2_ concentrations and ovarian response as observed by ultrasound. Once the leading follicles reached 14 mm in diameter, Cetrorelix (Merck-Serono, Germany) 0.25 mg subcutaneously was added and continued every day till day of hCG administration. In both groups, 10,000 IU of hCG (pregnyl, Daropakhsh, Iran) was administered IM when at least three follicles reached ≥18 mm in diameter. Ovum pickup was done by ultrasound-guided 36 hours after hCG injection.

**Semen processing before ICSI**

The semen samples were gathered by masturbation following 3-4 days of abstinence on the day of ovum pick up. Sperm concentration and total sperm count was assessed using a Makler chamber. Sperm motility and morphology analysis was performed under light microscopy according to the Kruger’s strict criteria and World Health Organization criteria (13, 14). Sperms with normal head in size, shape, and acrosome, without midpiece or tail defects were considered normal. After complete liquefaction, the semen was washed using density gradient sperm-separation techniques, and the final pellet was resuspended in 0.2 mL of medium. 

**Oocyte activation method**

After retrieval, oocytes were washed with G-MOPS medium (Vitrolife-Sweden) and incubated in culture medium (GIVF-plus; Vitrolife ,Sweden) then covered with mineral oil (Ovoil; Vitrolife) for 2 hours at 37^o^C, 6% CO_2_ and 5% O_2_. Cumulus cells removed mechanically by a 30-second exposure to Hyase medium containing 80 IU/mL of hyaluronidase (Vitrolife, Sweden). Denuded oocytes were then evaluated for nuclear status. 

Oocytes showing the release of the first polar body were considered mature and were selected for ICSI. Oocytes with single, three, or more pronuclei, immature, malformed, and postmature were eliminated from this research. Eppendorf micromanipulator mounted on a Nikon inverted microscope was used to perform ICSI. In control group (n=145 oocyte), routine ICSI was applied and oocytes transferred in the culture media for incubation at 37^o^C and 6% CO_2_ and 5% O_2_. 

In the artificial oocyte activation (AOA) group (n=168 oocyte) immediately after ICSI, oocytes were entered and incubated in culture medium containing 5 µM of calcium ionophore (A23187) (Sigma, St. Louis, MO, USA) for 5 minutes. Then injected oocytes washed at least five times with G-MOPS solution and incubated in the same culture medium. The oocytes were checked 16-18 h after injection to determine the presence of pronuclei using a Nikon inverted microscope. 

Activated oocytes were defined with observation of at least one pronucleus or cleaved oocytes. The oocytes were cultured in-vitro for 2-3 days to assess their development and cell division.

**Evaluation of embryo quality and embryo transfer**

Fertilization and embryo quality were assessed by a skilled embryologist. Cleavage stage embryos are graded according to the Hill’s criteria (15): Grade A was considered the high-quality embryo, without fragmentation, equal-sized homogenous blastomeres (4-cell embryo on day 2 or 8- cell embryo on day 3) and homogeneous cytoplasm. Grade B: embryos with ≤10% fragmentation, equal sized homogenous blastomeres. Grade C: embryos with ≤50% fragmentation, unequal sized blastomeres, and large granules. Grade D: embryos with >50% fragmentation, unequal sized blastomeres, and large black granules. 

Embryo transfer was performed by ultrasound guidance 48-72 hours after oocyte retrieval. For each couple, from one to three embryos were transferred, depending on the embryo quality and the age of the woman using a Labotect catheter (Labotect, Gotting, Germany). Remaining embryos were frozen in liquid nitrogen using verification method. All the patients received progesterone 100 mg/day, IM (Aburaihan Pharmaceutical Co., Tehran, Iran) and estradiol valerate 6 mg/day (Aburaihan Pharmaceutical Co., Tehran, Iran) as luteal support, starting on the day of oocyte retrieval.

**Outcome measures**

The primary outcome, including fertilization rate, chemical and clinical pregnancy rate were analyzed. The secondary outcomes, such as cleavage rate and percentage of high-quality embryos were also measured. The percentage of cleaved embryo was calculated as follow: total of cleaved embryos/number of zygotes Χ 100. Fertilization rate in both groups was calculated by the ratio of fertilized oocytes to the total number of survived injected metaphase II (MII) oocytes multiplied by 100. 

Serum βhCG concentration was measured two weeks after embryo transfer. After pregnancy confirmation, progesterone and estradiol valerate were continued until the tenth week of pregnancy. Chemical pregnancy was deﬁned as serum βhCG ≥25 IU/L measured 2 weeks after embryo transfer. Clinical pregnancy was documented by the presence of a gestational sac with heart beat on vaginal or abdominal ultrasound at 4-5 weeks after embryo transfer. 

The implantation rate was characterized as the fraction of gestational sac (s) to the number of embryos transferred. Clinical abortion rate was defined as clinically recognized pregnancy losses before 20 weeks of gestation.

**Statistical analysis**

The SPSS 19 software package was used to perform all the statistical analyses. The normality of distribution of variables was tested by using the Kolmogorov-Smirnov test. Independent sample T-test was used for continuous variables which were normally distributed and Mann-Whitney U test for data not normally distributed. Chi-squared test or Fisher exact test were used for qualitative variables as appropriate. p<0.05 was considered statistically significant. 

## Results

**Figure 1:**
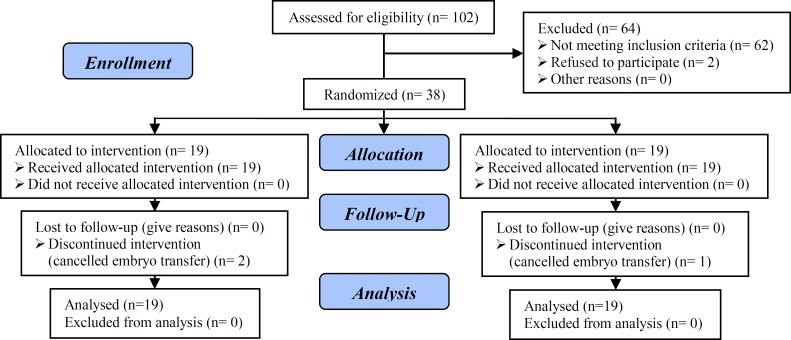
Recruitment follow-up and drop outs over the course of the study

The results were reported in accordance with the Consort statement. Of 102 couples candidates for ICSI, 38 patients were enrolled in our study. There was no patient lost to follow-up. In two patients in the AOA group and one patient in control group the embryo transfer were cancelled because of ovarian hyperstimulation syndrome. Although, the women who had to cancel the embryo transfer, were participated in the final analysis. The Consort statement flow diagram is presented in Figure 1.

Overall, 38 patients participated in this study. The patients were divided randomly into two groups as study and control groups (n=19 in each group). A total of 202 MΙΙ oocyte were obtained from the AOA group. Of them, 168 oocytes were selected for ICSI. Moreover, 145 out of 171 MΙΙ oocytes were selected in the control group. Basic and demographic characteristic of patients are shown in table I. The demographic parameters were similar in both groups, including male and female age, basal FSH level, type and duration of infertility. 

Table II shows descriptive information regarding semen parameters in AOA and control groups. There were no significant differences in sperm count, morphology, total and quick motility between groups. Table III presents a comparison of cycle characteristics between the two groups. As showed, there was no statistically significant difference in the number of oocytes retrieved and mature oocytes between two groups. Moreover, we found an insignificant difference in the endometrial thickness and the estradiol level in AOA group patients when compared to control group. The data of embryo scoring in both groups are listed in Table IV. The number and grade of transferred embryos, as well as, the number of frozen embryos in AOA group showed no significant differences when compared to the control group. In the AOA group, 35.3% of embryos were grade C. In contrast, 5.6% of embryos in the control group were graded as C, although the difference was not significant (p=0.08). Interestingly, there was a significantly difference in the number of embryos obtained and fertilized oocytes between two groups (p=0.04 in two instances).

Table V shows pregnancy outcome in the two groups. The statistical analysis of the data revealed that there was no difference between the two groups in regard to cleavage rate. However, implantation and fertilization rates were insignificantly higher in AOA group when compared to control group. While, there were no significant differences in chemical, clinical and ongoing pregnancy rates between two groups; there was a trend toward increased in these variables in AOA group. Multiple pregnancies and spontaneous abortion rate were similar in two groups. Patients in AOA group were also subdivided based on sperm morphology in semen samples. So, the clinical pregnancy rate was compared between the subgroups. Our results showed that the clinical pregnancy rate in patients with sperm morphology <4% was 25%; whereas patients with sperm morphology between 4-10%, and 11-13%, had the clinical pregnancy rate about 37.5% and 60%, respectively. 

Indeed, oocyte activation in patients with normal sperm morphology between 4-13% showed a beneficial effect on pregnancy outcome. It seems that the patients with sperm morphology <4% do not get benefit from oocyte activation.

**Table I:** Basic and demographic characteristics of patients

**Characteristics**		**Study group (N= 19)**	**Control group (N= 19)**	**p-value**
Male age (years) (Mean ± SD)		34.63 ± 3.94	35.1 ± 5.98	0.77
Female age (years )(Mean ± SD)		28.82 ± 3.35	29.94 ± 4.51	0.39
Basal FSH (IU/L) (Mean ± SD)		6.06 ± 1.9	6.89± 2.36	0.24
Duration of infertility (years) (Mean ± SD)		6.52 ± 3.9	6.39± 5	0.92
Infertility kind				0.66
	Primary [n (%)]	15 (78.9%)	17 (89.5%)	
	Secondary [n (%)]	4 (21.1%)	2 (10.5%)	

Study group: artificial oocyte activation .Student T-test, Mann-Whitney test, Fisher’s exact test, and Chi-square test. p-value<0.05 was significant.

**Table II:** Semen parameters in two groups

**Parameter**	**Study group (N= 19)**	**Control group (N= 19)**	**p-value**
Sperm count/ml (Mean ± SD)	19.75 ± 15.38	21.73 ± 20.94	0.74
Total motility (Mean ± SD)	15.3 ± 13.89	19.52 ± 15.88	0.39
Quick motility (Mean ± SD)	2.05 ± 3.23	1.31 ± 2.66	0.38
Normal morphology			0.75
Normal morphology (<4%) [n (%)]	4 (21.1%)	5 (26.3%)	
Normal morphology (4-10%) [n (%)]	9 (47.4%)	10 (52.6%)	
Normal morphology (11-13%) [n (%)]	6 (31.6%)	4 (21.1%)	

Study group: artificial oocyte activation. Student *t*-test and Chi-square test. p-value<0.05 was significant

**Table III:** Cycle characteristics

**Characteristics**	**Study group N=19**	**Control group N=19**	**p-value**
Follicles ≥16 mm No.	11.57 ± 2.96	10.73 ± 3.98	0.46
Oocytes retrieved No.	10.63 ± 5.26	9.1 ±4.12	0.32
Total mature oocyte No.	8.63 ± 4.63	7.57 ± 3.48	0.45
Injected oocyte MII No.	6.73 ± 3.31	5.57 ± 2.24	0.21
Endometrial thickness on day of hCG injection (mm)	8.42 ± 0.99	8.86 ± 1.41	0.27
Peak E_2_ on day of hCG injection (pg/ml)	1441.42 ± 879.19	1605.94 ± 688.19	0.52

Data are presented as Mean±SD.

p-value<0.05 was significant.

Study group: artificial oocyte activation. Mann-Whitney test and student *t*-test.

**Table IV:** Embryo data of the study and control group

**Characteristics**		**Study group (N= 19)**	**Control group (N= 19)**	**p-value**
No. of embryos obtained (Mean ± SD)		5.80 ± 3.39	4 ± 2.16 (*4.5)	0.04
No. of fertilized oocytes (Mean ± SD)		6.36 ± 3.02	4.57 ± 2.14 (*5)	0.04
No. of embryos freeze (Mean ± SD)		2.22 ± 3.57	2.22 ± 2.01	0.2
No. of embryos transferred (Mean ± SD)		2.58 ± 0.5	2.44 ± 0.7	0.49
Grade of transferred embryos				0.08
	A [n (%)]	6 (35.3%)	8 (44.4%)	
	B [n (%)]	6 (29.4%)	9 (50%)	
	C [n (%)]	6 (35.3%)	1 (5.6%)	
	D [n (%)]	0 (0%)	0 (0%)	

Study group: artificial oocyte activation. Student t test and Chi-square test. *Median-p<0.05 was significant.

**Table V:** Pregnancy outcome

**Characteristics**	**Study group (N= 19)**	**Control group (N= 19)**	**p-value**
Fertilization rate	95.33%	84.4%	0.11
cleavage rate	89.56%	87.74%	0.76
Implantation rate	17.64%	7.4%	0.14
Chemical pregnancy rate/cycle [n (%)]	8 (47.1%)	3 (16.7%)	0.07
Clinical pregnancy rate/cycle [n (%)]	7 (41.2%)	3 (16.7%)	0.14
Ongoing pregnancy rate [n (%)]	6 (85.7%)	3 (100%)	1
Clinical abortion rate [n (%)]	2 (11.8%)	0 (0%)	0.22

Study group: artificial oocyte activation. Chi-square test. P-value<0.05 was significant

## Discussion

Despite great advances in clinical and laboratory aspects of intra-cytoplasmic sperm injection (ICSI) procedure, fertilization failure still occurs in 2-3% of ICSI cycles (16). One of the reasons for failed fertilization after ICSI is lack of oocyte activation (4, 7, -). In these cases, various mechanical, electrical and chemical methods are known to use for artificial oocyte activation. Among these methods, chemical oocyte activation was currently used with substances such as calcium ionophore. In ICSI cycles, oocyte activation with calcium ionophore has been effective at increasing the fertilization rate (11, 19).

In the present study to improve outcomes of ICSI , 168 selected injected oocytes were chemically activated with calcium ionophore (A23187) immediately after ICSI. Our results showed that there was no significant difference in fertilization and cleavage rate between two groups. Implantation, chemical and clinical pregnancy rate was higher in the AOA group compared with control group; although the difference was not significant. The results of this study are in contrast with the results of Borges and coworkers (11) . They reported an improvement in ICSI outcomes following calcium ionophore oocyte activation in azoospermic patients. The researchers found that calcium ionophore improved ICSI outcomes only the epididymal, but not testicular spermatozoa, were injected. 

In another study, Nasr-Esfahani *et al *evaluated efficiency of ionomycin on ICSI outcomes in teratozoospermic patients . They reported that the fertilization and cleavage rate at 72 hours post-ICSI were significantly higher in study group. Moreover, there was no significant difference in cleavage rate 48 hours after ICSI and the percentage of high-quality embryo 48 and 72 hours after ICSI between the two groups. The overall pregnancy rate was reported 37% in this study (4). 

In a study by Eftekhar *et al*, the researchers analyzed the effect of artificial oocyte activation (AOA) on unfertilized oocytes 24 hours after ICSI using calcium ionophore (A23187) (5). In their study, fertilization and cleavage rate were72.5% and 62.7%, respectively. However high quality embryos remained low. According to a study in 2006 by Lu *et al*, exposure of unfertilized oocytes to a combination of calcium ionophore and puromycin could effectively activate them within 20-68 hour after ICSI (20). After 20 hours fertilization rate, cleavage rate and good quality embryos were 91%, 64% and 44% respectively. They detected passing of time after ICSI, resulted low fertilization and cell division rates. So time scale of exposure with calcium ionophore may affect ICSI outcome. Our study results were in agreement with Eftekhar *et al* and Lu *et al studies *(5, 20). These studies reported a similar fertilization rate in spite of different timing of oocyte activation after ICSI that demonstrating an optimal time of 24 h to expose the oocyte with calcium ionophpre. 

 In our research cleavage rate (89.56%) was comparable with the results reported by Nasr-Esfahani (74%); but was higher than Eftekhar study results (62.7%)(4), (5) . These discrepancies may be due to differences in the different timing of oocyte activation after ICSI. The results of our study indicated a no significant difference in the implantation, chemical and clinical pregnancy rates between two groups; however there was a trend toward increasing these variables in AOA group. Although some inconsistency was observed, but these findings show that artificial oocyte activation using calcium ionophore in ICSI cycles improve the implantation, chemical and clinical pregnancy rates (5, 11).

The percentage of good quality embryos in our research were lower than that reported by Nasr-Esfahani *et al* (35.3% vs. 70%) (6). It's known that some of the chemical agents can cause intracellular calcium elevation in the oocyte through single or multiple signaling pathways (21). So, there is no doubt that different chemical agent and duration of exposure can result in various ICSI outcomes after oocyte activation. To stimulate oocyte activation in the present work, we applied calcium ionophore (A23187) for 5 minutes; nevertheless, in the study of Nasr-Esfahani *et al*, the investigators have used Ionomycin for 10 minutes (4).

In addition, in Nasr-Esfahani *et al* study, Grade “A” and “B” embryos were considered high-quality embryos; but in our study, only Grade “A” embryos were defined as high-quality embryos (4). Our findings in this study showed a higher fertilization rate than those reported in Eftekhar *et al* study (95.33% vs. 72.5%), which is probably due to difference in duration time after ICSI and oocyte activation. In this study oocyte activation was done 24 hours and in our study immediately after ICSI. Oocyte aging is the most common cause of poor results in IVF/ICSI procedures (22). 

Therefore, the time interval between oocyte retrieval and fertilization is critical to obtain good quality embryos. Long term culture of oocyte results in zona hardening, increase the rate of parthenogenesis, disturbed embryo development, and poor embryo quality (23). In our investigation, abortion rate in AOA group was higher than control group; although, this rate was comparable with general population. This finding is in consistent with the prior researches that showed a normal chromosomal status in embryos derived through artificial oocyte activation (20).

## Conclusion

In this study, chemical oocyte activation with calcium ionophore resulted in an insignificant improvement in the implantation, fertilization, cleavage and pregnancy rates after ICSI. On the other, oocyte activation could significantly increase the number of fertilized oocytes and embryos obtained. Finally, calcium ionophore may improve ICSI outcomes in infertile men suffering from teratoospermia. Therefore further study with more patients is suggested to provide greater value.
